# B cell dysfunction in thalamus and brainstem involvement and high lactate caused by novel mutation of *EARS2* gene

**DOI:** 10.1186/s13052-025-01999-5

**Published:** 2025-05-19

**Authors:** Yu Wen, Yanmei Huang, Wendi Zhang, Ping Chen, Xiufen Hu, Xin Xiong, Li Luo

**Affiliations:** 1 Department of Pediatrics, Tongji Hospital, Tongji Medical College, Huazhong University of Science and Technology, Wuhan, China; 2 Department of Pathogen Biology, School of Basic Medicine, Tongji Medical College, Huazhong University of Science and Technology, Wuhan, China

**Keywords:** EARS2, Gene mutation, LTBL, B cell, BCR signal

## Abstract

**Purpose:**

The *EARS2* gene, a member of the mt-aaRS family, encodes mitochondrial glutamyl-tRNA synthetase (GluRS), which is involved in the synthesis of mitochondrial proteins. Pathogenic defects in EARS2 may cause mitochondrial OXPHOS deficiency, which is associated with a rare autosomal-recessive mitochondrial disease, leukoencephalopathy with thalamus and brainstem involvement and high lactate (LTBL).

**Methods:**

In this study, clinical features were obtained, and whole-exome sequencing was conducted on a patient with LTBL. B- and T-cell immunophenotyping and protein expression were analyzed using flow cytometry, and B-cell metabolism was investigated using confocal microscopy.

**Results:**

The patient with LTBL exhibited typical neurological manifestations, recurrent respiratory tract infections, and humoral immune disorders. Molecular analysis revealed a compound heterozygous novel mutation in c.1304T > A (p.L435Q) and a previously reported c.319 C > T (p.R107C) mutation of *EARS2*. The mutations led to protein structural modifications of EARS2. The patient also exhibited disrupted peripheral B-cell differentiation and B-cell receptor signal transduction. The *EARS2* mutation led to decreased expression of CD38 and dysfunction of mitochondrial metabolism, with elevated reactive oxygen species levels in B cells.

**Conclusion:**

We identified a novel mutation of the *EARS2* gene in a patient with LTBL, expanding the mutation database. The mutation of *EARS2* modified protein structure and impaired B-cell function, decreased CD38 expression, and led to dysfunction of mitochondrial metabolism, all of which may account for the recurrent respiratory tract infections and humoral immune disorders observed in LTBL.

**Supplementary Information:**

The online version contains supplementary material available at 10.1186/s13052-025-01999-5.

## Introduction

Mitochondria provide energy to cells via oxidative phosphorylation (OXPHOS). Besides the central function of supplying energy to cells, mitochondria also play essential roles in other important cellular functions, such as apoptosis, calcium homeostasis, inflammation, and immunity [1, 2]. Mitochondrial translation is important for proper mitochondrial function via the synthesis of respiratory chain complexes regulated by mitochondrial aminoacyl-tRNA synthetases (aaRSs). Mt-aaRSs, encoded by nuclear DNA (*aaRS2*), are key enzymes in protein synthesis and ensure efficient genetic code translation with high fidelity by catalyzing the covalent attachment of amino acids to their corresponding tRNAs [3]. Mutations of *aaRSs* lead to mitochondrial respiration chain complex dysfunction and cause many human diseases, primarily with neuropathy and myopathy, usually transmitted as autosomal recessive traits [4, 5].

*EARS2*, a member of the mt-aaRS family, encodes mitochondrial glutamyl-tRNA synthetase (GluRS), which is involved in the synthesis of mitochondrial proteins [6, 7]. Pathogenic defects in *EARS2* may cause mitochondrial OXPHOS deficiency, which is associated with the rare autosomal-recessive mitochondrial disease, leukoencephalopathy with thalamus and brainstem involvement and high lactate (LTBL). LTBL has a broad spectrum of clinical symptoms, including psychomotor retardation, hypotonia, and seizures, with similar cardinal neuroimaging magnetic resonance imaging (MRI) characteristics. For late-onset disease (usually after 6 months of age), patients display relatively mild symptoms, followed by clinical improvement, while for early onset disease (usually neonatal/early infantile), patients show more severe symptoms and a rapidly progressive course [8–10].

Herein, we report a novel mutation of *EARS2* in a patient with LTBL who experienced recurrent respiratory infection and exhibited disordered B cell function, suggesting a novel EARS2 function in the immune system.

## Materials and methods

### Clinical profile

The proband (G5P2), an infant with a 3.9 kg birth weight is the second child of healthy and non-consanguineous parents. The patient was born in the 39th week of gestation after a normal pregnancy and delivery. She could raise her head at 4 months. At follow-up, she had poor head control, was unable to sit or crawl, was unable to grab objects, and had no language skills. She had a depressed nasal bridge, orbital hypertelorism, and hand hemangioma and presented with hypotonia in all limbs. She was hospitalized with pneumonia in the neonatal period at 7 and 10 months of age (Moraxella catarrhalis and Haemophilus influenzae were detected respectively). Laboratory analyses of the blood revealed elevated serum lactate, pyruvate, creatine kinase, ammonia, and alanine aminotransferase levels and decreased immunoglobulin (Ig) levels (IgA 0.06 g/L and IgM 0.28 g/L).

### Genetic and protein analysis

Genetic analysis was performed after the consent from the parents of the patient. Whole exome sequencing of the whole blood was performed by Oumeng V Medical Laboratory (Wuhan, China). The gene and amino acid sequences were obtained from the GenBank database and UniProt database (https://www.uniprot.org/). The amino acid sequence alignment analysis was performed using DNAMAN software (http://www.lynnon.com/). The 3D structure of the protein was predicted using AlphaFold2, and the top-ranked structure was selected as the target protein. The mutant structure of the protein was constructed by PyMOL software (https://pymol.org/2/) and treated with energy minimization.

### Preparation of PBMCs

Peripheral blood was collected and human peripheral blood mononuclear cells (PBMCs) were isolated by density centrifugation using a lymphocyte separation solution for PBMCs preparation. The PBMCs were stored in liquid nitrogen until further use.

### Staining and flow cytometry

For B cell detection, PBMCs were stained with the following surface antibodies: Percp-anti-7AAD, FITC-anti-CD19, PE-anti-CD24, APC-anti-CD27, BV510-anti-IgD, and PB-anti-CD38. Naïve B cells (CD19^+^CD27^low^IgD^high^), memory B cells (CD19^+^CD27^high^IgD^low^), transitional B cells (CD19^+^CD38^high^CD24^high^), and plasmablasts (PBC; CD19^+^CD38^high^CD24^low^). B cells were identified from PBMCs.

For T cell detection, PBMCs were stained with the following surface antibodies: PerCP-anti-CD62L, APC-Cy7-anti-CD3, APC-anti-CD4, FITC-anti-CD8, PE-Cy7-anti-CCR7, BV605-anti-CD45RA, Pacific Blue-anti-CD38. TCM (CD45RA^low^CCR7^high^), TN (CD45RA^high^CCR7^high^), TEM (CD45RA^low^CCR7^low^), and TEMRA (CD45RA^high^CCR7^low^). T cells were identified in CD3^+^CD4^+^ or CD3^+^CD8^+^ PBMCs.

For mitochondrial mass detection, PBMCs were stained with FITC-anti-CD19, and then incubated with biotin-F(ab′)_2_ anti-human Ig (M + G) at 37℃ for 5 min. The cells were stained with PK-mito dye for 5 min.

Data were collected on a BD flow cytometer (BD Biosciences, CA, USA) and analyzed using FlowJo X 10.0.7r2 software (BD Biosciences, CA, USA).

### Phosphorylated flow

PBMCs were incubated with FITC-anti-CD19 and biotin-F(ab′)_2_ anti-human Ig (M + G) (10 µg/mL) on ice for 30 min. The cells were activated in a 37 ℃ water bath for 5 min and then fixed, permeabilized, and stained with anti-BAFFR, anti-CD79a, anti-pSyk, anti-pY, anti-pCD19, anti-pBtk, anti-CD86, anti-pPI3K, anti-pmTOR, and anti-pWASp antibodies (Abclonal, Wuhan, China). Data were collected using a BD flow cytometer and analyzed using FlowJo software.

### Western blot

Total protein was extracted from PBMCs, and western blot was performed using a previously described protocol [11]. Blots were incubated with rabbit anti-EARS2 (Cusabio, Wuhan, China) and mouse anti-GAPDH (Proteintech, Wuhan, China) primary antibodies.

### Confocal microscopy

PBMCs were incubated with FITC-anti-CD19 on ice for 30 min, and subsequently incubated with biotin-F(ab′)_2_ anti-human Ig (M + G) at 37℃ for 5 min, followed by DHE or PK-mito dye for 5 min. Images were obtained on a Nikon confocal fluorescence microscope, and data were analyzed using the NIS-elements AR 5.01.

### Statistical analysis

GraphPad Prism software was used to perform the statistical analysis and two-tailed unpaired t-test was performed to assess the statistical significance. *P* < 0.05 suggested a statistically significant difference.

## Results

### Neurological alterations in a patient with *EARS2* mutation

**Fig. 1:**
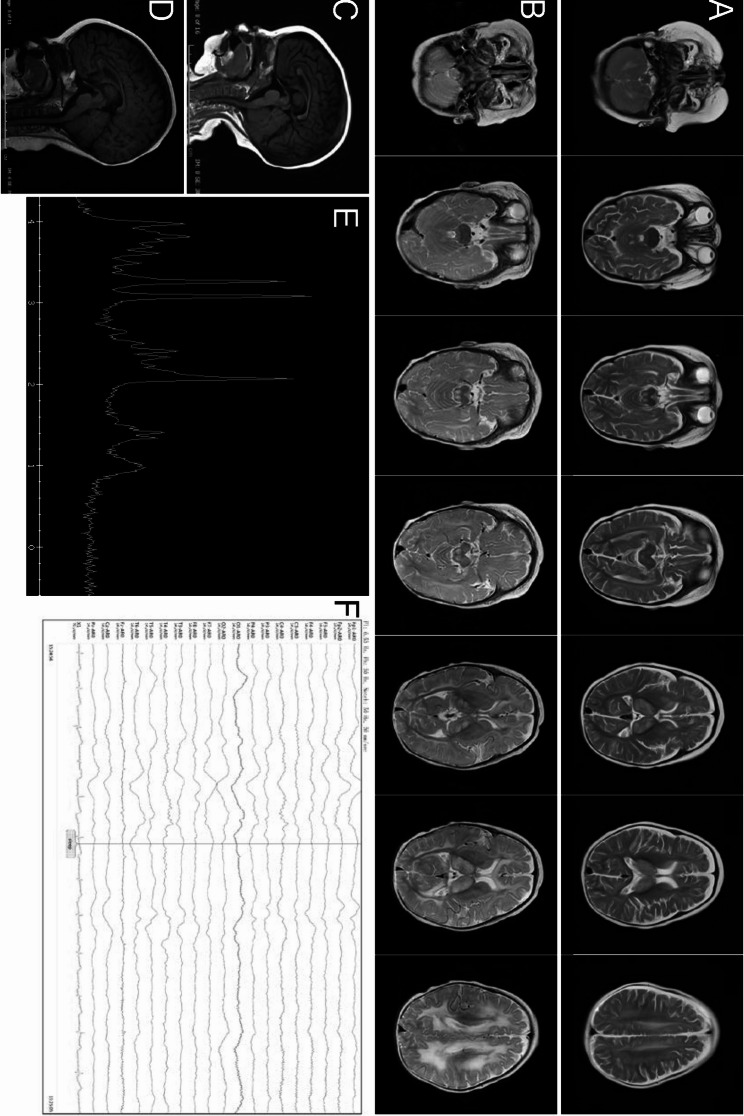
*EARS2* mutation leads to neurological alterations. Magnetic resonance imaging (MRI) images of our patient acquired at 7 months of age (**a**,** c**) and 10 months of age (**b**,** d**), showing bilateral and symmetrical abnormal long signals on T2-weighted images of the dorsal thalamus, head of caudate nucleus, brain stem, cerebellar hemispheres, and frontal, parietal, and occipital white matter areas. Hypogenesis of the corpus callosum is shown **(c**,** d).** An elevated lactate peak is observed in the short TE MR spectrum **(e).** Diffuse background slow waves (1.5–3 Hz) are shown on electroencephalography **(f)**

MRI of the brain revealed symmetrical T1 hyperintense and T2 hyperintense signals in the bilateral dorsal thalamus, head of the caudate nucleus, brainstem, bilateral cerebellar hemispheres, and subcortical regions of the frontal, occipital, and parietal lobes at 7 months of age (Fig. 1a, c). Demyelination of the white matter was worse at 10 months old than at 7 months (Fig. 1b, d). Besides an elevated lipid/lactate peak at 1.3 ppm, an increased choline peak and a decreased N -acetyl aspartate peak were detected in the thalamus region (Fig. 1e). Abnormal EEG revealed large multifocal amounts of spikes, sharp waves, slow waves, and sharp slow waves. Spikes and sharp waves were obvious in the bilateral parietal areas, particularly on the left side (Fig. 1f).

### A novel conservative mutation of *EARS2*

**Fig. 2:**
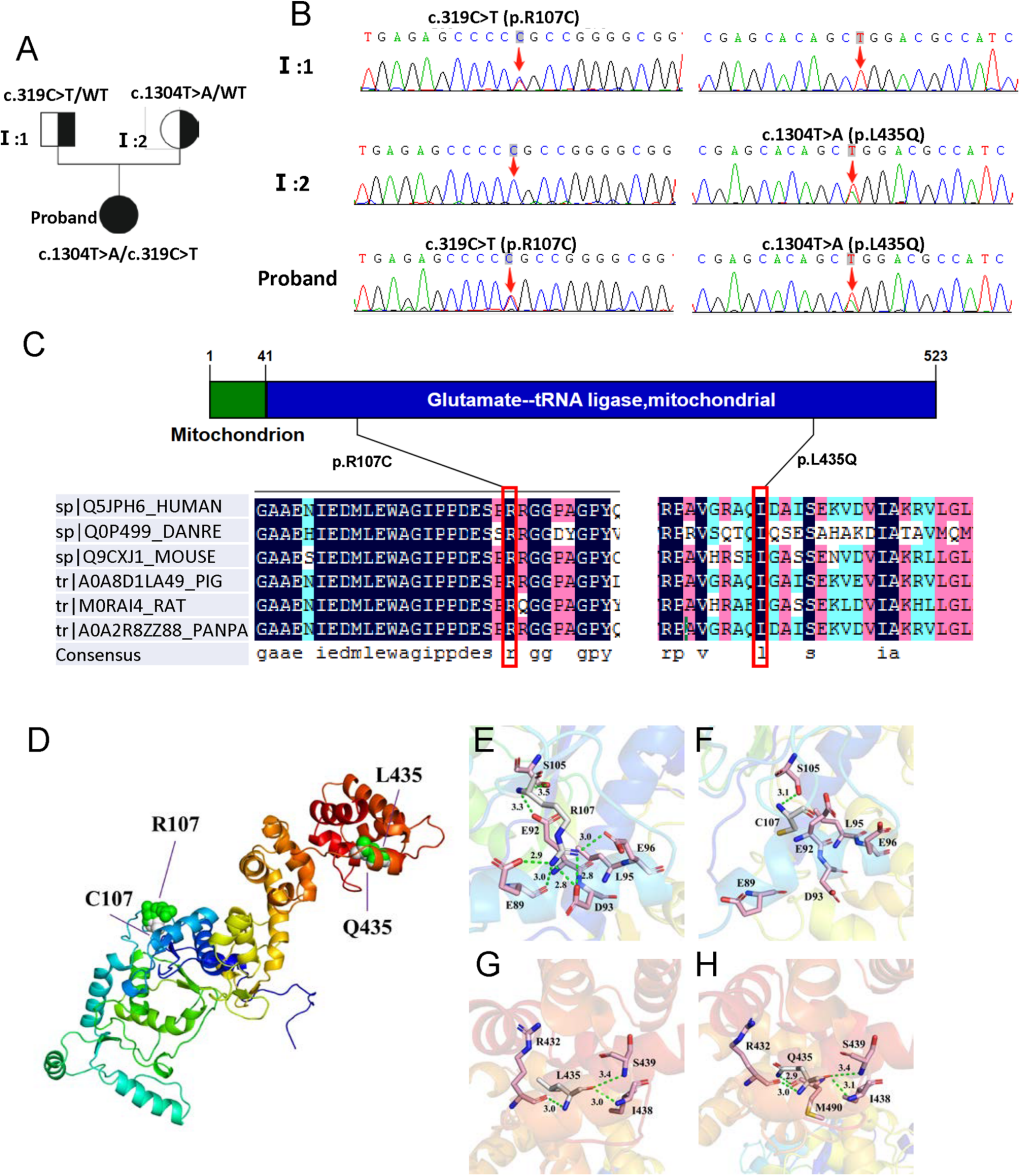
Pedigree of the families and mutation analysis of c.319 C > T (p.R107C)/c.1304T > A (p.L435Q) mutations in the *EARS2.***(a)** Family pedigree. **(b)** Father of the proband with the heterozygous p.R107C mutation and mother of the proband with the heterozygous p.L435Q mutation. Probands with p.R107C and p.L435Q mutations. **(c)** Conservation analysis revealed that arginine at position 107 and leucine at position 435 are highly conserved in different animal species and human EARS2 proteins. Arrow indicates the mutation location. **(d)** Superimposed structure of wild-type EARS2 protein (green) and R107C/L435Q variant (white color). **(e-h)** Changes in local environment and polar residue distance of EARS2 protein caused by the R107C and L435Q mutations. The residual structures of wild-type R107 (**e**) and L435 (**g**) protein are shown. The R107C (**f**) and L435Q **(h)** variants are shown. Green dotted lines, hydrogen bonds

Whole exome sequencing of the patient and her parents was performed. The results revealed two compound heterozygous variants of *EARS2* (NM_001083614.2): c.319 C > T (p.R107C) and c.1304T > A (p.L435Q) in exons 3 and 7, respectively. The results were verified using Sanger sequencing. DNA analysis confirmed that both parents were heterozygous carriers of the mutation; her mother had the c.1304T > A (p.L435Q) mutation and her father had the c.319 C > T (p.R107C) mutation (Fig. 2a, b). The c.319 C > T (p.R107C) mutation has been reported before [12], while the c.1304T > A (p.L435Q) mutation has not been previously reported in disease databases (ClinVar, BIC) or in the literature, suggesting a novel mutation site of *EARS2*. The complete amino acid sequences of EARS2 protein of human (sp|Q5JPH6), Danio rerio (sp|Q0P499), Mouse (sp|Q9CXJ1), Pig (tr|A0A8D1LA49), Rat (tr|M0RAI4), and Pan paniscus (tr|A0A2R8ZZ88) were obtained from the UniProt database and the amino acid sequence alignment analysis showed high conservation of amino acid residue at 107 and 435 among multiple species (Fig. 2c). Further, to predict the effect of these mutations on protein structure, we established a protein modeling. An analysis of the model 3D structure revealed changes in protein conformation (Fig. 2d). The R107C and L435Q mutations modified the number of hydrogen bonds and changed their interactions with the surrounding amino acid residues (Figs. 2e–h). Thus, the protein structural modifications of EARS2 are caused by the c.319 C > T (p.R107C)/c.1304T > A (p.L435Q) mutations.

### *EARS2* mutation alters the B cells differentiation and BCR signaling

**Fig. 3:**
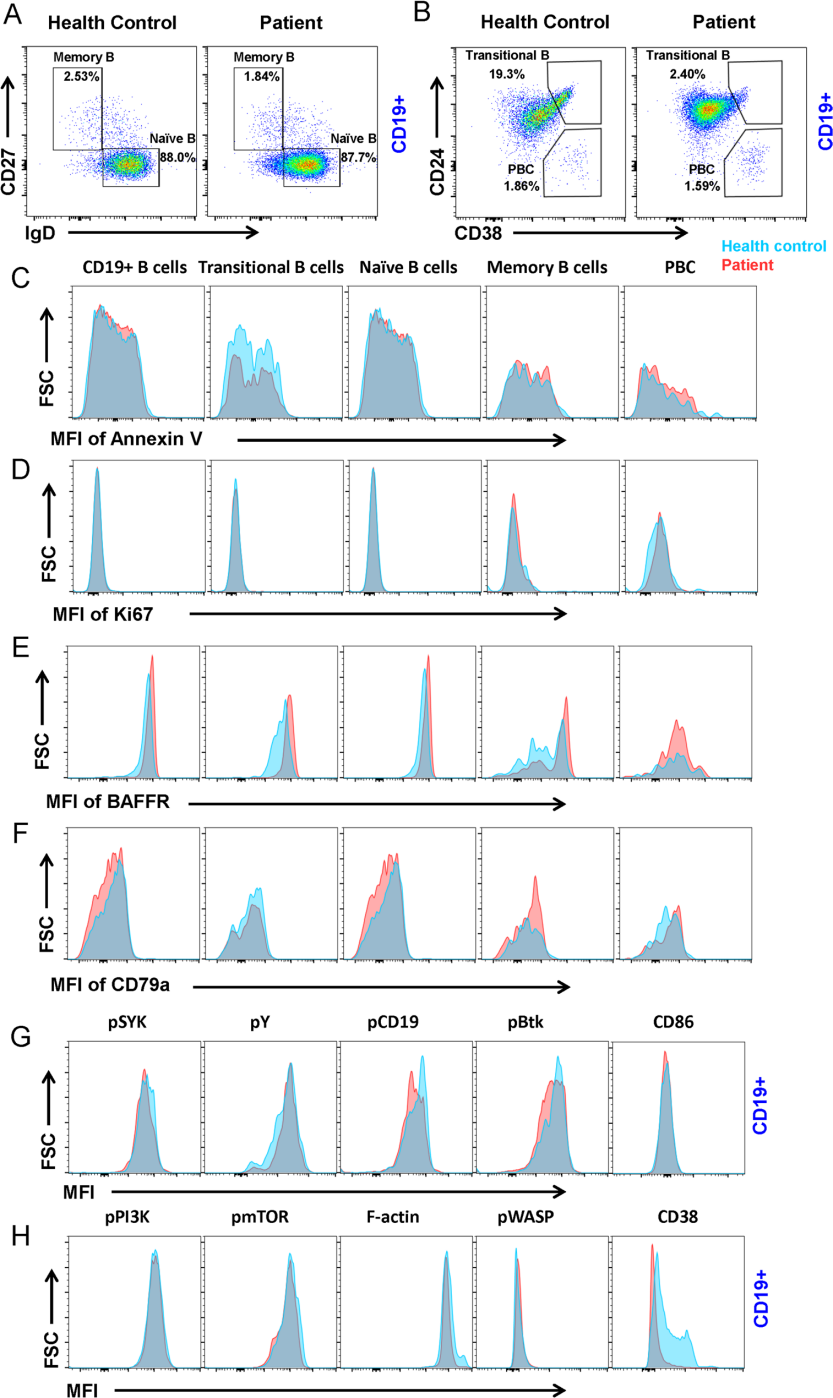
*EARS2* mutation leads to B cell alterations. **(a**,** b)** Analysis of CD19 + B cell subsets from human peripheral blood mononuclear cells (PBMCs) from the patient with EARS2 mutation and healthy control. Memory B cells (CD27 + IgD-), naïve B cells (CD27-IgD+), transitional B cells (CD24 + CD38+), and PBCs (CD24-CD38+) were gated from CD19 + B cells. **(c**,** d)** Analysis of the MFI of Annexin V and Ki67 of B-cell subsets. **(e, f)** Analysis of the MFI of BAFFR and CD79a of B-cell subsets. **(g**,** h)** The levels of pSYK, pY, pCD19, pBtk, CD86, pPI3K, pmTOR, F-actin, pWASP, and CD38 of CD19 + B-cells

The proband had a humoral immune disorder and experienced recurrent respiratory tract infections. To further investigate the effect of the mutation of *EARS2* on immune function, flow cytometric analysis was used to identify different B-cell subsets in PBMCs. Naïve B cells and memory B cells were distinguished by staining for CD27 and IgD, whereas transitional B cells and PBCs were distinguished by staining for CD24 and CD38. We found no significant differences in the percentages of naïve B cells, memory B cells, or PBCs between healthy controls and the proband (Fig. 3a, b). Interestingly, there was a decrease in the percentage of transitional B cells in the patient compared to the healthy control (Fig. 3b). No significant changes were observed in the proliferation or apoptosis of B cell subsets by Ki67 and Annexin V staining (Fig. 3c, d).

BAFF receptor (BAFFR) is expressed on the surface of most B cells and is an important pro-survival receptor [13]. We found that BAFFR expression increased in CD19^+^ total, transitional, naïve, and memory B cells (Fig. 3e). CD79a, a major B-cell receptor (BCR) component, plays an important role in B-cell development in the bone marrow and B-cell maturation [14]. We found that the expression of CD79a was decreased in CD19^+^ B cells and naïve B cells and increased in memory B cells and PBCs (Fig. 3f). Upon antigen stimulation, the initiation and transduction of BCR signaling were controlled by phosphorylation of the immunoreceptor tyrosine-based activation motif (ITAM) domains of CD79a/CD79b and the recruitment of SYK, BLNK, and BTK (Fig. 3g). We found that the phosphorylation of the co-stimulator CD19 decreased in the patient with *EARS2* mutation. Moreover, pSyk and pBtk decreased in the proband’s B cells, whereas pPI3K, pmTOR, and pWASP did not change significantly (Fig. 3h). CD38 is a B-cell surface receptor that plays an important role in human immune regulation by regulating B cell activation, proliferation, and differentiation [15]. Our results revealed a significant decrease in CD38 expression in CD19^+^ B cells (Fig. 3h). These data indicate that EARS2 mutation leads to B cell differentiation and BCR signal transduction and alteration via CD38.

### EARS2 mutation led to dysfunction of B cell metabolism

**Fig. 4:**
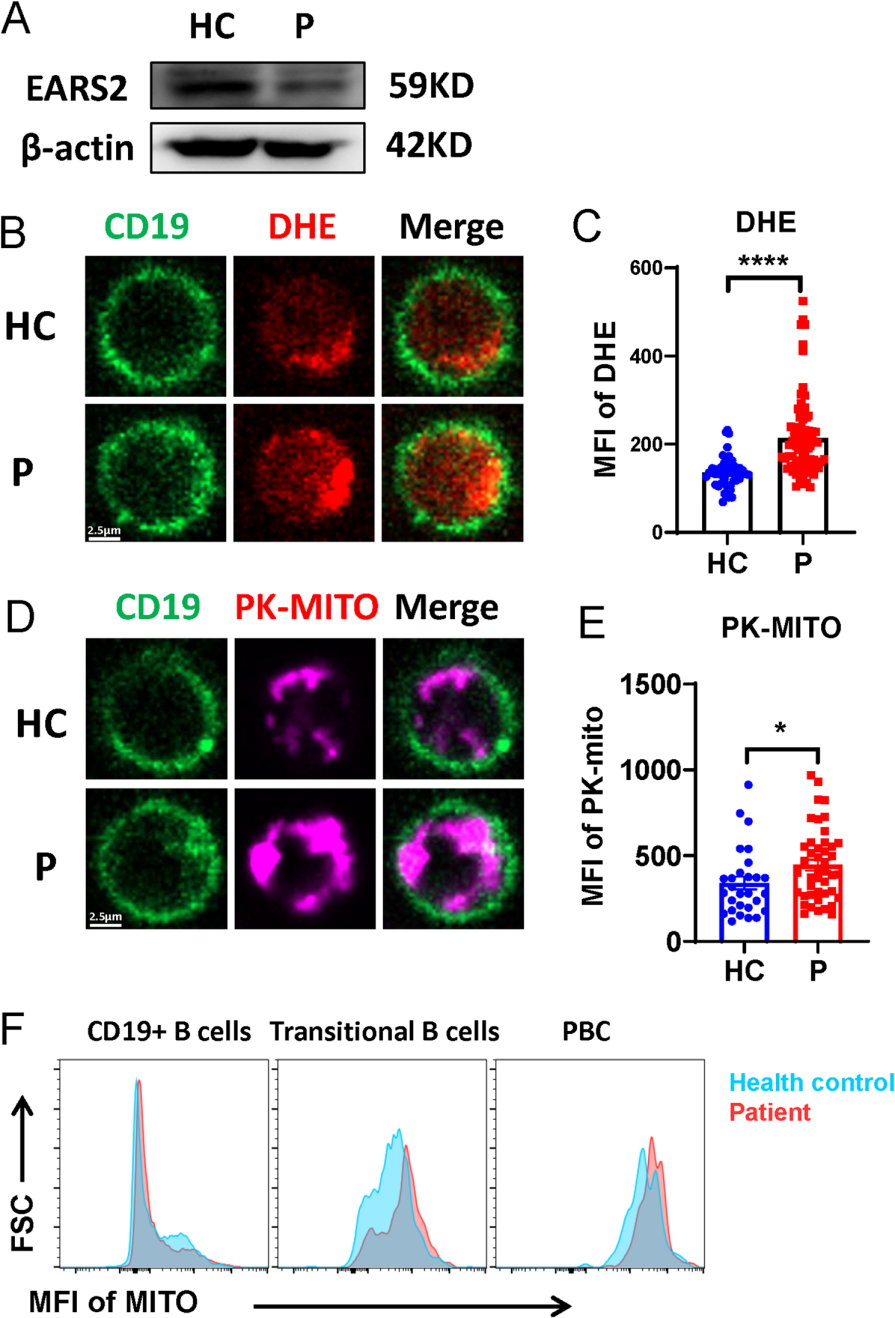
*EARS2* mutation alters B-cell metabolism. **(a)** The EARS2 level of PBMCs was analyzed by western blot. **(b–e)** The levels of ROS and mitochondrial mass of B cells were analyzed by confocal. **(f)** MFI of mitochondria of B cell subsets were analyzed by FCM

Metabolism is crucial for B-cell proliferation and differentiation. B cell activation and BCR signal transduction are regulated by metabolism [16]. Glutamyl-tRNA synthetase 2, which is encoded by *EARS2*, is required for mitochondrial protein synthesis. Mutations in *EARS2* lead to dysfunction in mitochondrial oxidative phosphorylation. We further investigated whether the mutation of *EARS2* causes metabolic dysfunction in B cells. EARS2 protein expression was decreased in the proband, as verified by western blotting (Fig. 4a). We found that the level of reactive oxygen species (ROS) was increased in the activated B cells from the patient with the *EARS2* mutation (Fig. 4b, c), indicating a dysfunction of total oxidative phosphorylation. Moreover, confocal microscopy indicated a higher mitochondrial mass in EARS2 mutated B cells (Fig. 4d, e). Interestingly, transitional B cells and PBCs exhibited elevated mitochondrial mass in the patient with the EARS2 mutation (Fig. 4f).

### The defect of EARS2 led to T cell dysfunction

**Fig. 5:**
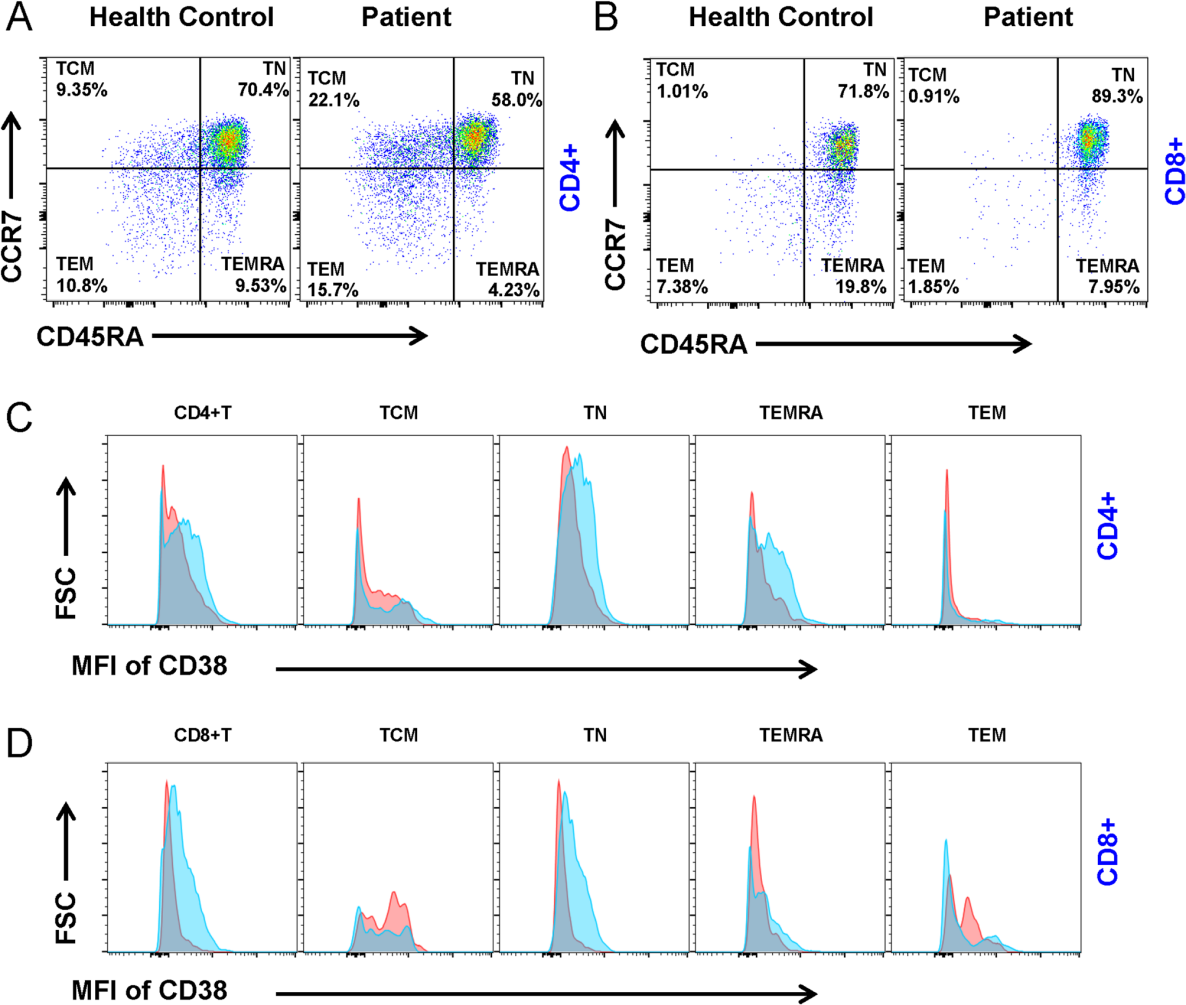
*EARS2* mutation alters T cells. **(a**,** b)** Analysis of CD4 + and CD8 + T cell subsets from PBMCs in the patient with EARS2 mutation and healthy control. Central Memory T cells (TCM) (CCR7 + CD45RA-), Naive T cells (TN) (CCR7 + CD45RA+), Effective Memory T cells (TEM) (CCR7-CD45RA-), and effector memory T cells re-expressing CD45RA (TEMRA) (CCR7-CD45RA+) were gated from CD4 + and CD8 + T cells. **(c)** The MFI of CD38 of CD4 + T-cell subsets were analyzed. **(d)** The MFI of CD38 of CD8 + T-cell subsets were analyzed

To test whether T cells were involved in the regulation of B cell function in the patient with *EARS2* mutation, we examined a subset of peripheral blood T cells. The proportions of TN and TEMRA CD4^+^ T cells were decreased, while the TCM and TEM populations were elevated in patients compared to the healthy control (Fig. 5a). We also examined the characteristics of the CD8^+^ T cell subsets. In the proband, the proportion of TEM and TEMRA were decreased compared to that in the healthy control (Fig. 5b). The expression of CD38 was also detected in T cells, with the results demonstrating that both CD4^+^ and CD8^+^ T cells showed low expression of CD38; for example, CD4^+^ TN, TEMRA, and CD8^+^ TN cells showed low levels of CD38 (Fig. 5c).

## Discussion

Mutations in the *EARS2* gene are associated with LTBL. Our results revealed compound heterozygous variants of EARS2 namely, c.319 C > T (p.R107C) and c.1304T > A (p.L435Q). The variant c.1304T > A (p.L435Q), a novel mutation in *EARS2* has not been described previously. The target amino acid of this mutation is highly conserved among species, suggesting that it plays an important role in protein synthesis. The R107C and L435Q mutations modified the hydrogen bonds and their interactions with surrounding amino acid residues that are necessary for maintaining protein structural integrity, leading to changes in the 3D structural conformation of EARS2 protein. The molecular structure analysis revealed the structural importance of positions 107 and 435. The structural changes induced by these gene mutations can affect protein function and lead to disease occurrence.

The clinical manifestations of LTBL are often characterized by multisystem damage, including brain, muscle, heart, kidney, and liver damage, with elevated serum lactate levels. The clinical severity of LTBL varies according to the abnormal mitochondrial rate [17]. Mild cases often show onset after 6 months of age and present with irritability and psychomotor regression. Improvements in clinical and biochemical indicators are often observed starting from the age of 2 years. Severe cases often present with early onset hypotonia, psychomotor retardation, seizures, and persistent lactate elevation [8, 9, 18]. Distinctive features of brain MRI in LTBL include bilateral and symmetrical T2 hypointense signals that appear in the deep white matter, thalamus, and brainstem [19].

Consistent with previous reports, early onset hypotonia and global developmental delay were observed in this case, with the MRI results showing extensive demyelination of the white matter, which had deteriorated at 10 months compared to 7 months. Interestingly, the patient experienced recurrent respiratory tract infections, including two cases of severe pneumonia that required treatment in the pediatric intensive care unit, providing clues to immune dysfunction.

Since the serum levels of Ig (IgM and IgA) were decreased in the patient, we first investigated B-cell function. Transitional B cells are immature B cells derived from the bone marrow following negative selection and are considered precursors of mature B cells that mediate antibody production and humoral immunity [20, 21]. We found a decrease in TrB in the patient with *EARS2* mutation, while no alteration in proliferation or apoptosis was observed in all B-cell subsets. BAFF plays an important role in B-cell selection and proliferation and promotes the differentiation of immature B cells into TrB cells [22]. Our results showed that the mutation of *EARS2* had no significant effect on BAFFR expression in B cells. BCR signaling is vital for sustaining normal B cell activation and differentiation. When the BCR is stimulated by specific antigens, it induces conformational changes and phosphorylation of ITAM domains, which recruit and phosphorylate BCR signaling proteins, such as LYN, Syk, Btk, and BLNK, to promote downstream signal conduction [23]. We further evaluated the expression of BCR signaling proteins to investigate B-cell function, and the results showed that the mutation of *EARS2* led to a decrease in the phosphorylation of Syk and Btk. Interestingly, we found that CD38 expression on the surface of B cells was significantly decreased in the patient with *EARS2* mutation. CD38 is a type II transmembrane glycoprotein with enzyme and signal transduction activities and is highly expressed in plasma blast cells, plasma blast cells, activated T cells, and B cells [24]. CD38 shows vital effects on B cell activation, proliferation, and differentiation by inducing tyrosine phosphorylation of Syk, PLC-γ, and Btk [15, 24]. Based on our results, we consider that the mutation of *EARS2* might negatively regulate B cell differentiation by inhibiting the expression of CD38, which positively regulates BCR signaling.

EARS2 participates in mitochondrial respiratory chain synthesis and regulates mitochondrial oxidative phosphorylation. In our study, the abnormal elevation of lactate and pyruvate of the patient indicated impaired mitochondrial OXPHOS when *EARS2* mutated. Dysfunction of EARS2 can damage multiple systems and organs. Elevated ROS levels and mitochondrial mass in *EARS2* mutated patient-derived fibroblasts have been observed compared to several control cell lines [25]. Moreover, our results showed increased ROS levels and mitochondrial mass in B cells from the patient with *EARS2* mutation compared to the control, showing that dysfunction of mitochondrial metabolism was associated with disturbed OXPHOS in *EARS2* deficient B cells. Based on these results, we assumed that the mutation of *EARS2* leads to B cell dysfunction by promoting ROS production due to disordered mitochondrial oxidative phosphorylation; however, this requires further research.

B and T cell interactions are critical for germinal center reactions and the generation of memory and plasma cells [24, 26]. We found that T cell differentiation was abnormal, and CD38 expression in CD4^+^ and CD8^+^ T cells was decreased in the patient, which might be associated with B cell dysfunction. Further in-depth research is required to clarify the effects of EARS2 on T cells.

There are still some potential limitations in this study. We reported one patient which is not sufficient to draw statistical conclusions. And it should be more accurate to assess OCR and ECAR by Seahorse to study OXPHOS dysfunction, which was not completed because of the too few cells.

Collectively, we report a novel mutation in *EARS2* in a patient with LTBL who presented with recurrent respiratory tract infections and a dysregulated immune system, which expands the mutation database. EARS2 dysfunction is induced by the protein structural modifications caused by the c.319 C > T (p.R107C)/c.1304T > A (p.L435Q) mutations. We reveal that the mutation of *EARS2* impairs B cell functions in terms of BCR signal transduction and B cell differentiation owing to the decreased expression of CD38 and dysfunction of mitochondrial metabolism.

## Electronic supplementary material

Below is the link to the electronic supplementary material.

Supplementary Material 1

## Data Availability

Data are available from the authors upon reasonable request and with permission of the Tongji Hospital, Tongji Medical College, Huazhong University of Science and Technology.

## Abbreviations

OXPHOS: Oxidative phosphorylation
aaRSs: Aminoacyl-tRNA synthetases
GluRS: Glutamyl-tRNA synthetase
LTBL: Thalamus and brainstem involvement and high lactate
MRI: Magnetic resonance imaging
Ig: Immunoglobulin
PBMCs: Human peripheral blood mononuclear cells
BCR: B-cell receptor
ITAM: Immunoreceptor tyrosine-based activation motif
ROS: Reactive oxygen species
TCM: Central Memory T cell
TN: Naive T Cell
TEM: Effective Memory T Cell
TEMRA: Effector memory T cells re-expressing CD45RA
