# Supplementary material for: B cell dysfunction in thalamus and brainstem involvement and high lactate caused by novel mutation of EARS2 gene
Source: Ital J Pediatr. 2025 May 19;51:143. doi: 10.1186/s13052-025-01999-5 (PMC12090667; doi:10.1186/s13052-025-01999-5)
Supplement: Supplementary file 1 — Supplementary Material 1 [file 13052_2025_1999_MOESM1_ESM.doc]

Li Luo, Ph. D

Department of Pediatrics,

Tongji Hospital, Tongji Medical College

Huazhong University of Science and Technology

Email: luoli001@126.com

Mar 5^th^, 2025

Dear Editors:

Please find enclosed our manuscript entitled “B cell dysfunction in thalamus and brainstem involvement and high lactate caused by novel mutation of EARS2 gene”, which we would like to submit for publication as a Research Ariticle in “***Italian Journal of Pediatrics***”.

The *EARS2* gene, a member of the mt-aaRS family, encodes mitochondrial glutamyl-tRNA synthetase (GluRS), which is involved in the synthesis of mitochondrial proteins. Pathogenic defects in EARS2 may cause mitochondrial OXPHOS deficiency, which is associated with a rare autosomal-recessive mitochondrial disease, leukoencephalopathy with thalamus and brainstem involvement and high lactate (LTBL). Whether the mutation of EARS2 causes immune system dysfunction remains unknown.

In our study, we first found a compound heterozygous novel mutation in c.1304T>A (p.L435Q) and a previously reported c.319C>T (p.R107C) mutation of EARS2. The mutations led to protein structural modifications of EARS2. The patient also exhibited disrupted peripheral B-cell differentiation and B-cell receptor signal transduction. The EARS2 mutation led to decreased expression of CD38 and dysfunction of mitochondrial metabolism, with elevated reactive oxygen species levels in B cells.

We believe that ***Italian Journal of Pediatrics*** is an ideal platform for disseminating our research due to its reputation for publishing high-quality and impactful work in the realm of immunology. The authors have no conflicts of interest to declare, and the research has been conducted in accordance with ethical guidelines and institutional review board approvals, where applicable.

Thank you for considering our submission. We look forward to the opportunity to contribute to the scholarly discourse in the field of immunology. Please feel free to contact me at luoli001@126.com if you require any additional information or have any questions regarding the manuscript. I am looking forward to your assessment of our manuscript.

Please address all correspondence to:

Dr. Li Luo, Department of Pediatrics, Tongji Hospital Affiliated to Tongji Medical College, Huazhong University of Science and Technology, Wuhan, China

We look forward to hearing from you soon.

Sincerely,

Dr. Li Luo
